# Immunotherapy for Breast Cancer: First FDA Approved Regimen

**DOI:** 10.15190/d.2019.4

**Published:** 2019-03-31

**Authors:** Georgiana R. Soare, Costin A. Soare

**Affiliations:** Hopital Femme Mere Enfant, Hospice Civile de Lyon, Lyon, France; QPathology, Boston, MA, USA

**Keywords:** Atezolizumab, Triple Negative Breast Cancer, Paclitaxel, combination, accelerated approval.

## Abstract

1 in 8 women will be affected by breast cancer, which is the most diagnosed malignancy among women. Although breast cancer was regarded as “immunologically cold”, recent studies demonstrate that immunotherapy can be successful employed in combination regimens for the treatment of triple negative breast cancer, an aggressive type of breast cancer without many treatment options available. In March 2019, the US Food and Drug Administration granted accelerated approval for the first immunotherapy-based regimen comprising atezolizumab in combination with protein-bound paclitaxel for patients with advanced metastatic TNBC, expressing programmed cell death-ligand 1 (PD-L1) and without previous systemic treatment for metastatic disease. This immunotherapy-based regimen is not only a promising therapy for the TNBC patients, but it also represents an inspiring proof of concept for the development of more efficient advanced immunotherapy-based strategies for breast cancer treatment in the future.

Breast cancer is the most diagnosed malignancy among women^1^. According to the Cancer Research Institute, in 2018 there were approximately 2.1 million new cases of breast cancer and 630,000 breast cancer-related deaths^2^, with ~ 270,000 breast cancer patients diagnosed and ~ 41,000 deaths in USA alone. Noteworthy, about 1 in 8 women will be affected by breast cancer in their lives^2^.

Most of the breast cancer risk factors are environmental or related to the personal lifestyle, estimated to be responsible for ~ 90% of the pathogenesis related to breast cancer development, while genetic predisposition may cause ~ 10%. The environmental and lifestyle factors include, but are not limited to, unhealthy eating habits, physical inactivity, reproductive factors (age of the menarche, age at first birth and age at menopause, breastfeeding, parity), hormonal therapy (e.g. Menopausal Hormone Therapy (MHT), which combines progestin and estrogen) alcohol and obesity^3^*.* While the lifestyle and environmental factors can be changed (e.g. healthy diet, exercise), most of the reproductive factors can’t. The inherited genetic mutations in breast cancer include the BRCA1 and BRCA2 mutations. These mutations increase the risk for developing breast cancer for woman before age 70 with about 45-65%^4^. 

Typical treatment for an early diagnosed breast cancer is surgery. Breast cancer surgery can be combined with targeted therapies (such as hormone therapy), chemotherapy and radiation treatment, depending on the stage of the breast cancer and specific molecular markers^2^. In particular, the triple negative breast cancers (TNBC) represents about 15% of all diagnosed breast cancers and it has not only the most aggressive pattern, but it is also very hard to treat. TNBC is characterized by the lack of the receptors for the estrogen and progesterone and of the human epidermal growth factor receptor 2 (HER2)^5^.

As previously mentioned by others, breast cancer is regarded as an “immunologically cold” type of cancer, which usually does not respond well to immunotherapy. However, recent studies now demonstrate that, immunotherapy can be successfully used in breast cancer treatment with improved results in combination regimens^2,6,7^. As such, the US Food and Drug Administration (FDA) granted accelerated approval in March 2019 to the first immunotherapy-based treatment regimen for the patients with breast cancer^8^. This regimen comprises atezolizumab (Tecentriq, Genentech Inc.) in combination with paclitaxel for patients with metastatic TNBC, expressing programmed cell death-ligand 1 (PD-L1) on their tumor cells (PD-L1 positive stain of immune cells found on ≥ 1% of the tumor area). As a companion diagnostic strategy, US FDA approved the Ventana PD-L1 test used in TNBC patients’ selection for this immunotherapy-based therapy^8^. This is important, since there is an acute need for treatment options for TNBC patients, TNBC being known for its ability to grow, invade and metastasize faster.

Atezolizumab is a PD-L1 inhibitor already approved for the treatment of a number of malignancies, including lung cancer (non-small cell lung cancer) and bladder cancer (urothelial cancer). PD-L1 is a ligand protein found on the surface of certain cancer cells which can block the immune system to mount an attack on them^2,6-8^. Paclitaxel is a mitotic inhibitor drug that stabilizes the microtubules and it is already commonly used in the clinic for breast cancer treatment^9^. Compared to other breast cancer subtypes, the TNBC is thought to be more immunogenic, with the tumor-infiltrating lymphocytes having a prognostic and predictive value^6^. Moreover, TNBC expresses a higher level of PD-L1 on its surface^6^. Thus, it is expected that PD-L1 inhibition may promote anti-tumor T-cell responses in this particular subtype of breast cancer^6^.

FDA approval was based on an interventional clinical trial (*NCT02425891*), called “A Study of Atezolizumab in Combination With Nab-Paclitaxel Compared With Placebo With Nab-Paclitaxel for Participants With Previously Untreated Metastatic Triple-Negative Breast Cancer (IMpassion130)”, randomized, double-blind and placebo-controlled, involving 902 women patients with metastatic and unresectable TNBC and without any prior systemic treatment for the metastatic disease. Randomized patients received intravenous infusions of either atezolizumab (840 mg) or placebo in combination with protein-bound paclitaxel (100 mg/m^2^) on days 1 and 15 (of every 28-day cycle) and days 1, 8, 15 (of every 28-day cycle) respectively^8^*.*

This clinical study showed promising results in progression-free survival, a measure of how long after the treatment initiation the patient lives without the cancer getting worse^10^. Median progression-free survival for the patients expressing PD-L1 on their breast cancer tumor cells receiving the combined treatment (atezolizumab plus paclitaxel) versus receiving placebo plus paclitaxel was 7.4 months versus 4.8 months. Stratified hazard ratio for progression free survival was 0.60 (95% CI: 0.48, 0.77; p<0.0001) in favor of the combined regimen with an objective response rate in subjects with confirmed responses of 53% in combined treatment, compared to 33% in placebo plus paclitaxel. Overall survival results were considered immature (43% deaths in the intent to treat population)^8^. Most common side effects observed in this study by using a combination of atezolizumab with protein-bound paclitaxel were**nausea, fatigue, alopecia, peripheral neuropathy, low blood counts, diarrhea, constipation, vomiting, cough, headache and decreased appetite^8,10^.

This immunotherapy-based therapy is not only a promising tool for the treatment of TNBC patients with limited treatment options, but it also represents a proof of concept that will lead to more efficient and advanced immunotherapy-based strategies for breast cancer treatment in the future.
